# Arabidopsis Heterotrimeric G-Proteins Play a Critical Role in Host and Nonhost Resistance against *Pseudomonas syringae* Pathogens 

**DOI:** 10.1371/journal.pone.0082445

**Published:** 2013-12-05

**Authors:** Seonghee Lee, Clemencia M. Rojas, Yasuhiro Ishiga, Sona Pandey, Kirankumar S. Mysore

**Affiliations:** 1 The Samuel Roberts Noble Foundation, Plant Biology Division, Ardmore, Oklahoma, United States of America; 2 Donald Danforth Plant Science Center, St. Louis, Missouri, United States of America; Leibniz-Institute for Vegetable and Ornamental Plants, Germany

## Abstract

Heterotrimeric G-proteins have been proposed to be involved in many aspects of plant disease resistance but their precise role in mediating nonhost disease resistance is not well understood. We evaluated the roles of specific subunits of heterotrimeric G-proteins using knock-out mutants of Arabidopsis Gα, Gβ and Gγ subunits in response to host and nonhost *Pseudomonas* pathogens. Plants lacking functional Gα, Gβ and Gγ1Gγ2 proteins displayed enhanced bacterial growth and disease susceptibility in response to host and nonhost pathogens. Mutations of single Gγ subunits Gγ1, Gγ2 and Gγ3 did not alter bacterial disease resistance. Some specificity of subunit usage was observed when comparing host pathogen versus nonhost pathogen. Overexpression of both Gα and Gβ led to reduced bacterial multiplication of nonhost pathogen P. syringae pv. *tabaci* whereas overexpression of Gβ, but not of Gα, resulted in reduced bacterial growth of host pathogen P. syringae pv. *maculicola*, compared to wild-type Col-0. Moreover, the regulation of stomatal aperture by bacterial pathogens was altered in Gα and Gβ mutants but not in any of the single or double Gγ mutants. Taken together, these data substantiate the critical role of heterotrimeric G-proteins in plant innate immunity and stomatal modulation in response to *P. syringae*.

## Introduction

Heterotrimeric guanine nucleotide-binding proteins (G-proteins hereafter) consisting of three distinct subunits, Gα, Gβ and Gγ, are conserved in all eukaryotes, and regulate a multitude of physiological processes [1–3]. In the inactive state, the Gα subunit binds guanosine diphosphate (GDP) and remains associated with the Gβγ dimer. External stimuli sensed by the cell surface-localized G-protein-coupled receptors (GCPR) trigger the activation of G-proteins by facilitating an exchange of guanosine triphosphate (GTP) for GDP, resulting in GTP-bound Gα and freed Gβγ dimer. Both these entities can interact with downstream targets of specific signal transduction pathways [1–4]. This cascade of events is stopped by the intrinsic GTPase activity of Gα that causes hydrolysis of the bound GTP resulting in GDP-bound Gα that re-associates with the Gβγ dimer [5]. In contrast to the multiplicity of G-protein complexes in animals where multiple genes exist for each subunit, the repertoire of plant G-proteins is relatively limited. Arabidopsis contains one Gα subunit encoded by *GPA1* [6], one Gβ subunit encoded by *AGB1* [7] and three Gγ subunits, Gγ1, Gγ2 and Gγ3, encoded by *AGG1*, *AGG2* and *AGG3*, respectively [8,9]. However, regardless of their fewer numbers, plant G-proteins play important roles in several signaling pathways, including plant immunity [3,8,10,11]. Moreover, little is known about the receptors that function upstream of heterotrimeric G proteins in the diverse biological processes, and canonical GPCRs have not been well-characterized in plants. A recent study described that multiple kinases are upstream of heterotrimeric G protein defense signaling for plant innate immunity [12]. 

Plant immunity involves several layers of defense that enable plants to recognize potential threats and mount the appropriate defense responses. Plants recognize the presence of potential pathogens by detecting common features present on pathogen surfaces and molecules resulting in microbe-associated molecular patterns or pathogen-associated molecular patterns (MAMPs/PAMPs), such as flagellin, lipopolysaccharide (LPS) and elongation factor Tu (EF-Tu). MAMP/PAMP recognition triggers a first layer of basal resistance resulting in PAMP-triggered immunity (PTI) [13]. Some pathogens are able to deploy effector proteins to counteract this first layer of defense and, consequently, trigger a second layer of plant defense resulting in effector-triggered immunity (ETI) [13], also known as gene-for-gene resistance, which occurs upon recognition of the pathogen effectors by the plant resistance proteins. Both PTI and ETI induce stomatal closure [14] and, in most cases, ETI triggers a rapid cell death called the hypersensitive response (HR); both responses limit access and multiplication of the pathogen [15]. Elicitation of the HR cell death is regulated by reactive oxygen species (ROS) [16].

Nonhost resistance is the most stable and a broad-spectrum plant defense against all isolates of a particular pathogen [17]. PAMP-induced defense plays an important role for nonhost resistance against *Pseudomonas syringae* strains [18,19]. PTI and ETI together contribute to nonhost resistance against various *Pseudomonas* pathogens [17,20]. It has been shown that glycolate oxidase (*GOX*), proline dehydrogenase (*ProDH1* and *ProDH2*), and squalene synthase (*SQS*) genes plays an important role in nonhost resistance through reactive oxygen species signaling, hypersensitive response and nutrient limitation, respectively [17,21,22]. 

Several studies have reported on the role of heterotrimeric G-proteins in nonhost resistance against fungal pathogens in Arabidopsis. Arabidopsis genes, *PENETRATION1* (*PEN1*), *PENETRATION2* (*PEN2*), and *PENETRATION3* (*PEN3*) have been identified as factors of pre-invasion resistance in response to non-adapted powdery mildew pathogens, *Blumeria graminis* and *Erysiphe pisi*, which in nature colonize grass and pea plants, respectively [23–27]. A recent study showed that Arabidopsis phospholipase *Dδ* (*PLDδ*) gene is involved in penetration resistance against barley powdery mildew fungus *B. graminis* f. sp. *Hordei*. Chemical inhibition of PLDs in *pldδ* mutant plants confirmed the specificity of this isoform alone in regulating penetration resistance [28]. Moreover, *AGB1* and Powdery Mildew Resistance 5 (PMR5) contribute to *PEN2*-mediated pre-invasion resistance to rice blast fungus *Magnaporthe oryzae* in Arabidopsis. However, other important plant defense factors such as RAR1 (required for Mla12 resistance 1), SGT1 (suppressor of the G2 allele of skp1) and NHO1 (nonhost 1), are not required for nonhost resistance against *M. oryzae* in Arabidopsis [29]. Furthermore, it is not known whether plant heterotrimeric G-proteins are involved in conferring nonhost resistance against P. syringae pathogens.

Several studies have reported a role for heterotrimeric G-proteins in plant immunity against host pathogens. In rice, the Gα subunit mutant (*rga1*) showed reduced resistance after inoculation with an avirulent race of *M. oryzae* [30]. In contrast, the Arabidopsis Gα mutant (*gpa1*) showed slightly increased resistance against necrotrophic fungal pathogens *Plectosphaerella cucumerina* and *Fusarium oxysporum* [10], while *agb1* (Gβ subunit mutant), *agg1* (Gγ1 subunit mutant) and *agg1-1c agg2-1* double mutant displayed increased susceptibility to *P. cucumerina*, *Alternaria brassicicola*, and *F. oxysporum* [10,11,31]. Moreover, *AGG1* but not *AGG2*, was shown to be induced after infection by *A. brassicicola* and *F. oxysporum* [31]. The defense responses initiated by G-proteins appear to involve the production of ROS with the concomitant onset of the HR. The rice *rga1* mutant showed reduced levels of hydrogen peroxide (H_2_O_2_), greatly reduced hypersensitive response and delayed induction of *PR* genes after elicitor treatment [30]. Similarly, silencing of Gα and Gβ in *Nicotiana benthamiana* caused reduced accumulation of H_2_O_2_, reduced HR and reduced expression of the defense genes *PR2b, EDS1, NbrbohA* and *NbrbohB* after treatment with the elicitor harpin in comparison with non-silenced control plants [32]. 

Recent studies have also shown that G-proteins are directly involved in regulation of stomatal aperture during defense response. In wild-type plants, the PAMP, flagellin (flg22), inhibits light-induced stomatal opening; *gpa1* mutants were shown to be impaired in this function and consequently stomata remained open after flg22 treatment [33]. Similarly, silencing of Gα, Gβ1 and Gβ2 in *N. benthamiana* inhibited elicitor-induced stomatal closure that was observed in non-silenced control plants [32]. 

Although there is strong evidence for the role of heterotrimeric G-proteins in plant defense after treatments with fungal pathogens and elicitors, the extent to which G proteins are involved in plant immunity against bacterial pathogens remains debatable. Previously, it was shown that Arabidopsis Gα (*gpa1-4*) and Gβ (*agb1-2*) mutants did not exhibit a differential response against virulent or avirulent strains of *P. syringae*, leading to the conclusion that the response to *P. syringae* was independent of heterotrimeric G-proteins [11]. However, three recent studies describe the role of Arabidopsis G-proteins in defense responses via NADPH oxidase mediated signaling pathways [34], *Mildew Resistance Locus O* (*MLO*)-mediated defense signaling [35], PTI [12], and *BIR1* (*BAK1-interacting receptor-like kinase1*)-mediated plant defense responses [12]. 

We have addressed these discrepancies by performing a comprehensive analysis of the role of G-proteins in bacterial resistance by using single and higher order G-protein mutants, in the context of both host and nonhost *P. syringae* pathovars. Our results clearly demonstrate, using various combinations of methods and mutants, that Gα, Gβ and Gγ are required for both host and nonhost resistance against adapted and nonadapted bacterial pathogens, respectively.

## Results

### Mutations of heterotrimeric G-protein Gα, Gβ and Gγ subunits impair both host and nonhost resistance against *Pseudomonas syringae* pathovars

To determine whether G-protein subunits Gα, Gβ and Gγ are involved in both host and nonhost resistance, we inoculated wild-type Col-0, *gpa1-4* (Gα mutant), *agb1-2* (Gβ mutant), *gpa1-4 agb1-2* (GαGβ double mutant), *agg1-2* (Gγ1 mutant), *agg2-2* (Gγ2 mutant), *agg1-1c agg2-1* (Gγ1Gγ2 double mutant) and *agg3-2* (Gγ3 mutant) plants with a host pathogen *P. syringae* pv. *maculicola*, an avirulent strain P. syringae pv. *maculicola* (*AvrRpm1*), and nonhost pathogens P. syringae pv. *tabaci* and P. syringae pv. *phaseolicola*. 

**Figure 1:**
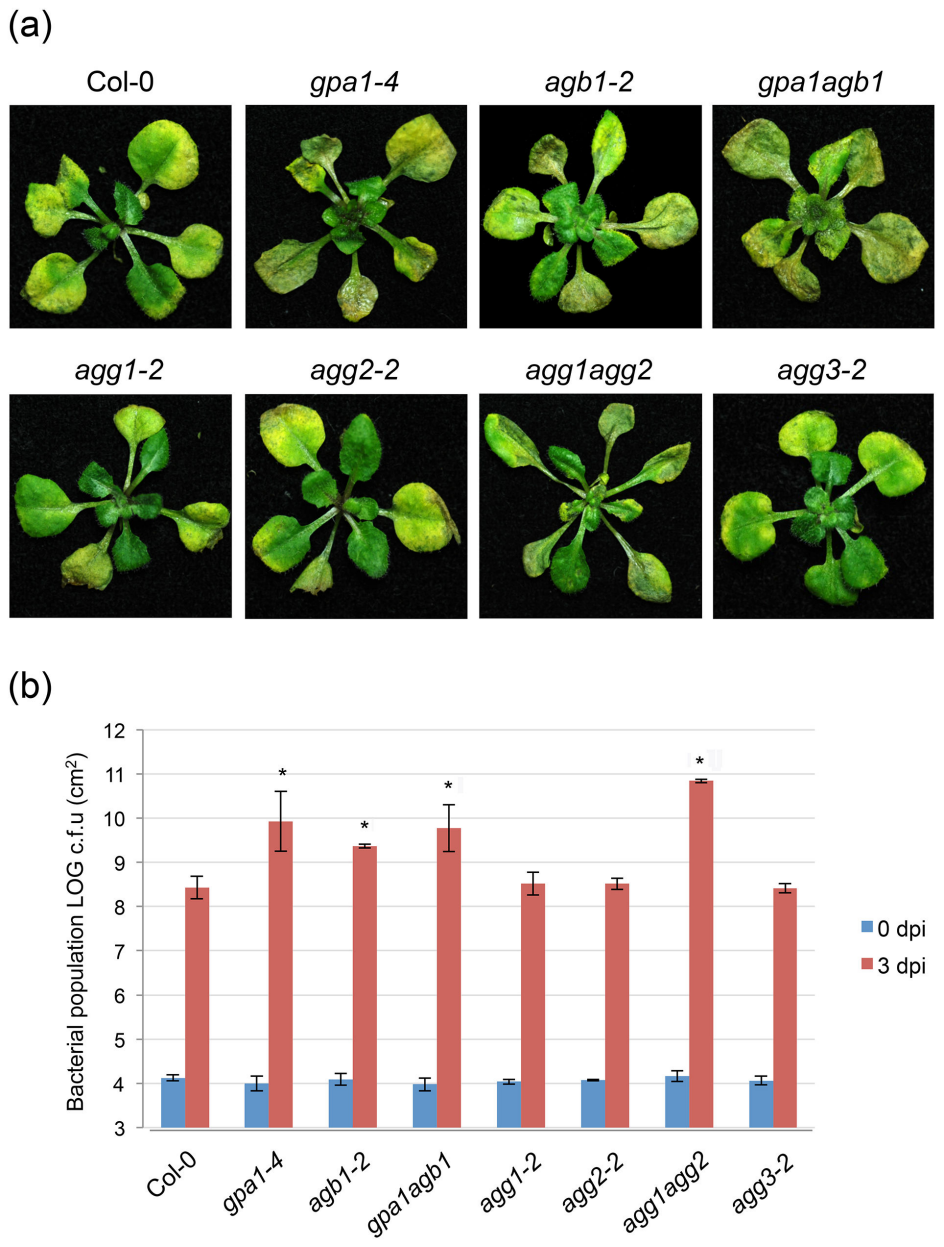
Disease symptoms and bacterial growth after flood-inoculation with the host pathogen P. syringae pv. *maculicola*. Two-week-old plants grown in 1/2 strength MS under short-day conditions (8 hrs of daylight) were flood-inoculated with the host pathogen *P. syringae* pv. *maculicola* at 3×10^6^ CFU/ml. (a) Disease symptoms in wild-type (Col-0) and heterotrimeric G-protein mutants (*gpa1-4*, *agb1-2*, *gpa1-4* *agb1-2* , *agg1-2*, *agg2-2*, *agg1-1c* *agg2-1* and *agg3-2*). Images were taken at 4 dpi. (b) Growth of *P. syringae* pv. *maculicola* in Col-0, *gpa1-4*, *agb1-2*, *gpa1-4* *agb1-2* , *agg1-2*, *agg2-2*, *agg1-1c* *agg2-1* and *agg3-2*. Bacterial titers at 0 and 3 dpi were measured by taking leaf disks from four inoculated plants. All experiments were independently repeated three times, and each experiment was performed with four replications. Bars represent average and standard deviations from all experiments. Asterisks above bars represent statistically significant differences in comparison with wild-type plants using Student’s *t*-test (P<0.05).

Three days after flood-inoculation with the host pathogen *P. syringae* pv. *maculicola*, *gpa1-4* and *agb1-2* plants showed enhanced disease susceptibility (Figure 1A) and 10-15-fold increase in bacterial growth in comparison to wild-type (Col-0) plants (Figure 1B). The *gpa1-4 agb1-2* double mutant also displayed enhanced disease symptoms and a similar increase in bacterial growth as the single mutant, *gpa1-4* (Figure 1B). In contrast to Gα and Gβ mutants, none of the single Gγ subunit mutants (*agg1-*2, *agg2-2* and *agg3-2*) showed any significant difference in disease susceptibility and bacterial growth in comparison to wild-type (Figure 1). However, the *agg1-1c agg2-1* double mutant showed significantly enhanced disease symptoms and ~20-fold increase in bacterial growth when compared to wild-type plants (Figure 1). 

**Figure 2:**
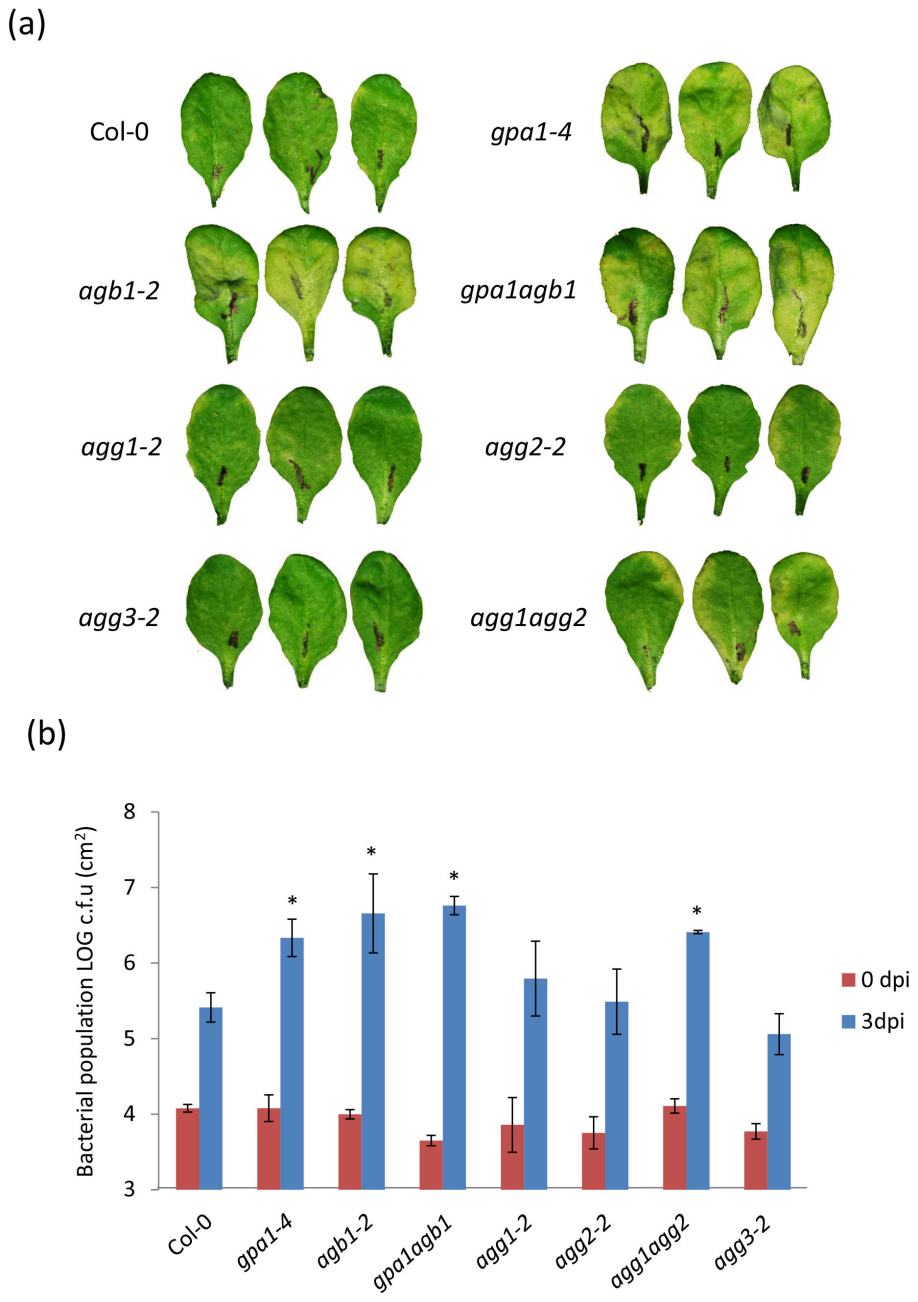
Disease symptoms, bacterial growth and accumulation of avirulent bacterial strain P. syringae pv. *maculicola* (AvrRpm1) in G-protein mutants. Leaves from 6-week-old plants were syringe-infiltrated with the avirulent pathogen *P. syringae* pv. *maculicola* (AvrRpm1) at 2.8 x 10^6^ CFU/ml. (a) Disease symptoms in wild-type (Col-0) and heterotrimeric G-protein mutants (*gpa1-4*, *agb1-2*, *gpa1-4* *agb1-2* , *agg1-2*, *agg2-2*, *agg1-1c* *agg2-1* and *agg3-2*). Images were taken three days after inoculation. (b) Growth of the avirulent pathogen *P. syringae* pv. *maculicola* (AvrRpm1) in Arabidopsis mutants. Leaf samples for bacterial quantification were taken at 0 and 3dpi. Bars represent mean and standard deviation for four biological replicates from each experiment. Two independent experiments were performed. Asterisks above bars represent statistically significant differences in comparison with wild-type plants using Student’s *t*-test (P<0.05).

We also checked the role of G-protein subunits in gene-for-gene resistance. Syringe-inoculation with a low concentration of avirulent pathogen P. syringae pv. *maculicola* (*AvrRpm1*) did not cause disease symptoms in the wild-type Col-0 plants due to the presence of the *RPM1* resistance gene [36]. Even though *RPM1* is present and expressed in all mutants, inoculation with the avirulent pathogen caused varying degrees of chlorotic disease symptoms. Specifically, the *gpa1-4*, *agb1-2*, *gpa1-4 agb1-2* and *agg1-1c agg2-1* mutant plants exhibited a 10-15 fold increase in bacterial growth after 3 dpi, in comparison with wild-type plants, suggesting that gene-for-gene resistance was impaired in these mutants (Figure 2). Bacterial growth in the single Gγ subunit mutants, *agg1-2*, *agg2-2* and *agg3-2*, was not significantly different from the levels reached in wild-type plants and no disease symptoms were observed in these mutants. 

**Figure 3:**
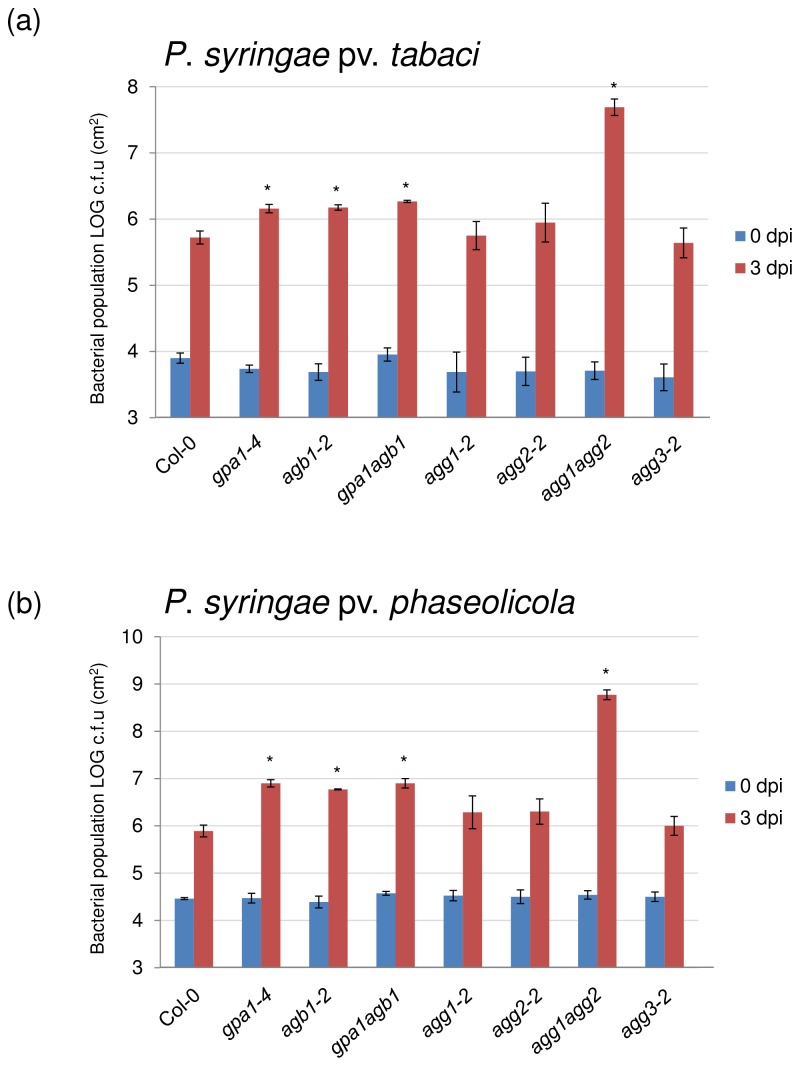
Measurement of bacterial growth after inoculation with nonhost pathogens P. syringae pv. *tabaci* and P. syringae pv. phaseolicola in Col-0, *gpa1-4*, *agb1-2*, *gpa1-4* *agb1-2* , *agg1-2*, *agg2-2*, *agg1-1c* *agg2-1* and *agg3-2*. Bacterial growth after flood-inoculation of P. syringae pv. *tabaci* (a) and P. syringae pv. phaseolicola (b) Two-week-old plants grown in 1/2 strength MS under short day conditions (8 hrs of daylight) were flood-inoculated with the nonhost pathogen *P. syringae* pv. *tabaci* at 3×10^6^ CFU/ml and P. syringae pv. phaseolicola at 7×10^6^ CFU/ml. Bacterial titers at 0 and 3 dpi were measured by taking leaf disks from four inoculated plants. All experiments were independently repeated three times, and each experiment was performed with four replications. Bars represent average and standard deviations from all experiments. Asterisks above bars represent statistically significant differences in comparison with wild-type plants using Student’s *t*-test (P<0.05).

We further evaluated the role of G-proteins in nonhost resistance using P. syringae pv. *tabaci*. As expected, no disease symptoms were observed in wild-type plants, however, flood-inoculation with this pathogen caused modest disease symptoms in *gpa1-4*, *agb1-2* and *gpa1-4 agb1-2* mutants (data not shown), consistent with a slight increase in bacterial growth (Figure 3A). The Gγ subunit double mutant *agg1-1c agg2-1* developed disease symptoms consistent with a dramatic increase (approximately 100-fold) in bacterial growth (Figure 3A), whereas no differences were observed in disease phenotypes or bacterial growth patterns in single Gγ subunit mutants compared to wild-type plants. 

To further verify the impairment of nonhost disease resistance in *gpa1-4, agb1-2, gpa1-4 agb1-2* and *agg1-1c agg2-1* mutant plants, we used another nonhost pathogen, *P. syringae* pv. *phaseolicola*. As expected, no symptoms were observed in wild-type plants, however, modest cell death was observed in *gpa1-4*, double mutants *gpa1-4 agb1-2* and *agg1-1c agg2-1* (data not shown). In addition, slightly higher levels of *P. syringae* pv. *phaseolicola* population (~ 10-fold) was observed at 3 dpi in *gpa1-4*, *agb1-2* and *gpa1-4 agb1-2* mutants compared to wild-type plants (Figure 3B). Strikingly, the *agg1-1c agg2-1* double mutant showed ~1000-fold increase in bacterial levels in comparison with wild-type plants (Figure 3B). Taken together, our results establish that the heterotrimeric G-protein subunits Gα, Gβ and Gγ play a critical role in plant innate immunity against *P. syringae*. Moreover, the Gγ3 subunit does not seem to be involved in regulating defense responses, whereas, both Gγ1 and Gγ2 are required to exhibit an effect. Interestingly, in these experiments, the *agg1-1c agg2-1* double mutant displayed higher disease susceptibility to both host and nonhost pathogens compared to *gpa1*, *agb1* single and *gpa1-4 agb1-2* double mutants. 

### Arabidopsis mutants of G-protein subunits are defective in stomatal closure in response to nonhost pathogen *P. syringae* pv. *tabaci*

**Figure 4:**
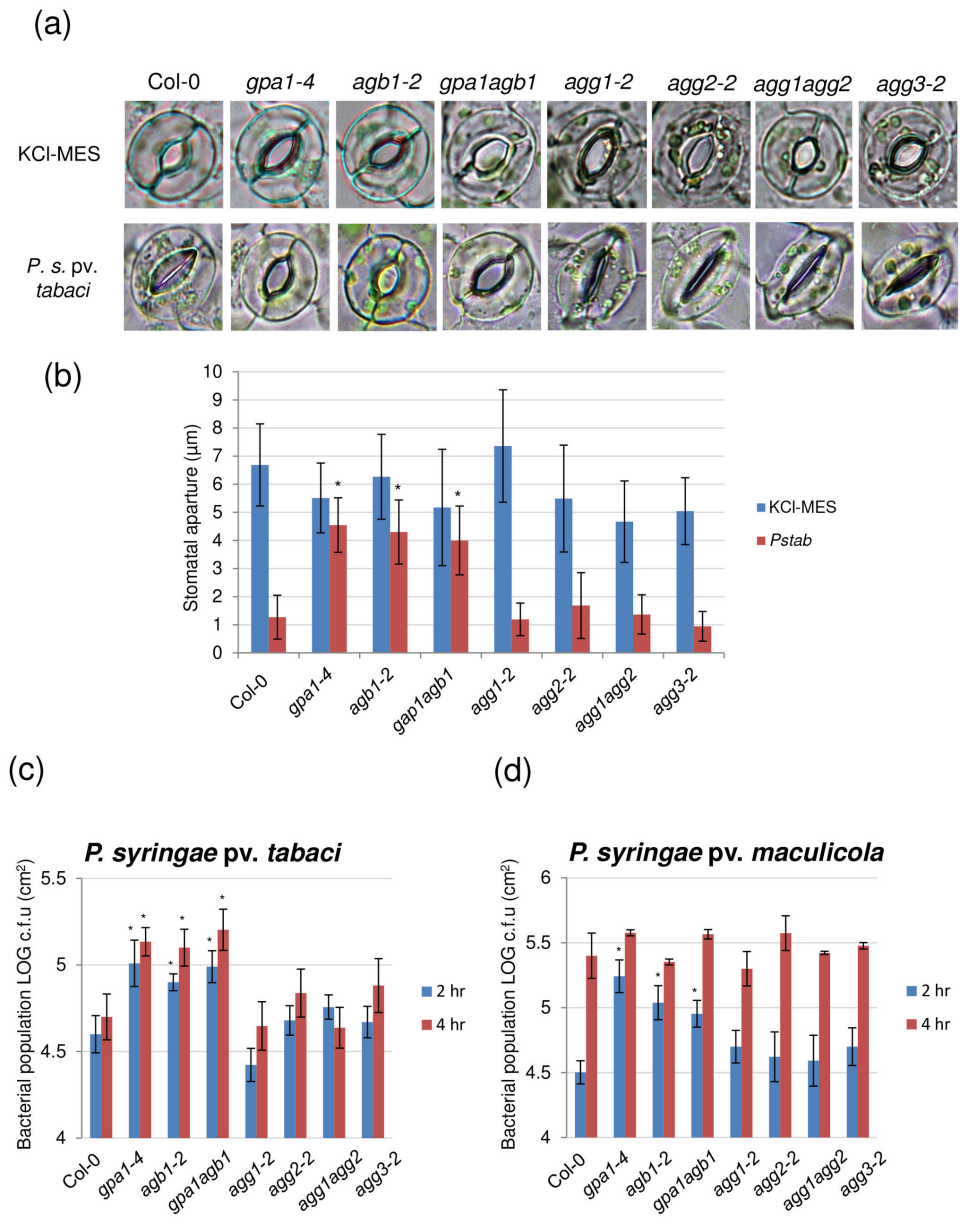
Stomatal closure and number of bacterial cells that entered through stomata after inoculation of nonhost pathogen P. syringae pv. *tabaci*. (a) Determination of stomatal closure and (b) aperture size induced by P. syringae pv. *tabaci* in epidermal peels. Stomatal aperture size was examined 2 hrs after P. syringae pv. *tabaci* inoculation. The epidermal peels prepared from all wild-type (Col-0) and heterotrimeric G-protein mutants were incubated in stomata opening buffer (KCl-MES) for at least 3 hrs to assure fully open stomata. Images were taken under a light microscope. Approximately 150 stomata were examined with five epidermal peel samples for each experiment. (c) Detached Arabidopsis leaves were floated on nonhost pathogen P. syringae pv. *tabaci* and (d) host pathogen P. syringae pv. *maculicola* (1.2 × 10^9^ CFU/ml). Detached leaf samples were collected 2 and 4 hrs after incubation and treated with 10% bleach for surface sterilization. The number of bacterial cells in the apoplast was determined. Data shown are means ± standard deviation (error bars) from four replicates per each experiment. Two independent experiments were performed with similar results. Asterisks above bars represent statistically significant differences in comparison with wild-type using Student’s *t*-test (P<0.05).

Stomata play a critical role in plant immunity by actively limiting the entry of plant pathogens [37]. There is one report of the *gpa1* mutant exhibiting insensitivity in flg22-induced inhibition of stomatal opening [33]; however, a comprehensive characterization of G-proteins for their function in stomatal closure in response to bacterial pathogens has not been performed. We therefore examined the stomatal closure of all the available Arabidopsis heterotrimeric G-protein mutants in response to the nonhost pathogen P. syringae pv. *tabaci* (Figure 4A).

Stomatal apertures of wild-type and G-protein mutants were measured after P. syringae pv. *tabaci* treatments. Two hrs after inoculation, the average width of the stomatal aperture was drastically decreased in the wild-type epidermal peels, whereas stomata remained open in *gpa1-4, agb1-2* and *gpa1-4 agb1-2* mutants (Figure 4B). It has been shown that stomata in *gpa1* and *agb1* are properly closed under dark treatment, and ABA-mediated inhibition of stomatal opening is impeded in the mutants [38,39]. We also observed a number of closed stomata in the *gpa1-4* and *agb1-2* mutants before incubating epidermal peels in stomata opening buffer. These findings suggest that stomatal opening and closing in the mutants is fully functional without stimuli such as ABA, dioleoyl-PA and PAMPs. Interestingly, none of the Gγ mutants tested showed defects in stomatal closure, similar to what has been reported for the abscisic acid (ABA)-induced stomata closure in Gγ mutants [8,40]. Together, these data demonstrate that Gα and Gβ subunit mutants are impaired in pathogen-induced stomatal closure. 

To directly measure the number of bacterial cells that entered through stomata, detached *Arabidopsis* leaves were floated on bacterial suspensions and bacterial cell numbers were quantified in the apoplast. After 2 hrs of inoculation with host (*P. syringae* pv. *maculicola*) or nonhost pathogen (P. syringae pv. *tabaci*), the number of bacterial cells inside *gpa1-4, agb1-2* and *gpa1-4 agb1-2* mutant leaves was significantly higher than in wild-type leaves (Figure 4C and D). The number of bacterial cells in *agg1-2*, *agg2-2*, *agg3-2* and *agg1-1c agg2-1* was not significantly different from that of wild-type. After 4 hrs of incubation, the number of bacterial cells in the leaf apoplast of wild-type plants was much higher in P. syringae pv. *maculicola* infected leaves than in P. syringae pv. *tabaci* infected leaves, likely due to the fact that P. syringae pv. *maculicola* produces the virulence factor COR to reopen stomata [41]. As a result, after 4 hpi, the quantity of P. syringae pv. *maculicola* bacterial cells in leaf apoplast of all G-protein mutants was similar to that of wild-type leaves (Figure 4D), whereas the number of P. syringae pv. *tabaci* cells continued to increase in *gpa1* and *agb1* mutants, but not in wild-type leaves. All together, these results suggest that heterotrimeric G-protein subunits Gα and Gβ, but not Gγ, play an essential role in mediating the stomatal closure in response to the nonhost pathogen P. syringae pv. *tabaci* in Arabidopsis. 

The genes encoding heterotrimeric G-protein are induced by host and nonhost pathogens, and the role of these genes in plant innate immunity is independent of the SA-mediated defense pathway

**Figure 5:**
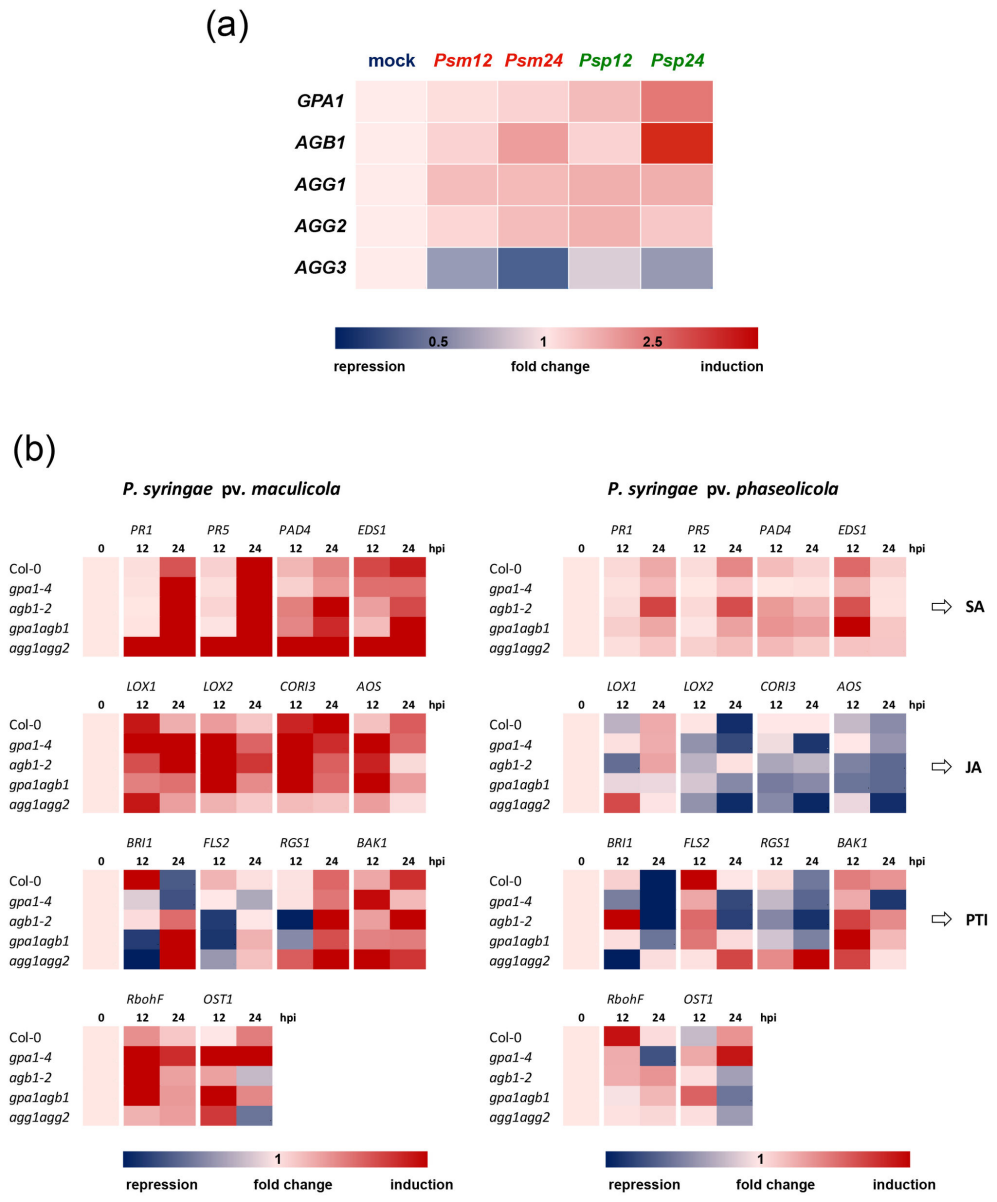
Heat map of transcript accumulation of heterotrimeric G-protein and defense related genes (SA, JA and FLS2 signaling pathways) regulated in response to P. syringae pv. *maculicola* and P. syringae pv. phaseolicola infections. (a) Expression of GPA1, AGB1 AGG1, AGG2 and AGG3 in wild-type upon P. syringae infection. Seedlings grown on 1/2 strength MS medium were inoculated with bacterial pathogens. The gene expression levels were determined 12 and 24 hrs after host, P. syringae pv. *maculicola* (Psm), and nonhost pathogen, P. syringae pv. phaseolicola (Psp), inoculations. (b) Gene expression profiling of various defense-related genes in G-protein mutants in comparison to wild-type (Col-0) plants. The expression of SA and JA defense-related genes and genes involved in *FLS2*-mediated defense signaling were examined after 12 and 24 hrs (hpi) in response to host, P. syringae pv. *maculicola*, and nonhost pathogen, P. syringae pv. phaseolicola. Each column is the fold change of gene expression as determined by qRT-PCR at 12 and 24 hpi in pathogen-inoculated samples. The relative gene expression values normalized by *Ubiquitin5* (UBQ5) and *Elongation* *factor 1* *alpha* (EF1α) were represented as n-fold compared to the mock-treated plants. Red and blue indicate up-regulated and down-regulated expression levels, respectively.

The transcript levels of all heterotrimeric G-proteins were induced after both host and nonhost pathogen inoculations except for the level of *AGG3* transcript, which was down-regulated (Figure 5A). We confirmed this observation by examining the publically available Arabidopsis expression database (Arabidopsis eFP Browser, http://bar.utoronto.ca/welcome.htm). This analysis shows that consistent with our data, the expression of *GPA1*, *AGB1, AGG1* and *AGG2* is up-regulated by *P. syringae* pathogens and bacterial PAMPs (Flg22 and HrpZ), further supporting the involvement of heterotrimeric G-protein-mediated pathways in the regulation of PTI and ETI. 

Salicylic acid (SA) is the major plant defense hormone in response to biotrophic plant pathogens (those that require a living host) such as *P. syringae*. Jasmonic acid (JA) signaling is mutually antagonistic with the SA-mediated defense pathway [42–44]. In order to evaluate the function of these signaling pathways in the G-protein-mediated defense responses, we measured the expression levels of key genes involved in SA and JA signaling pathway in wild-type and G-protein mutants, *gpa1-4*, *agb1-2*, *gpa1-4 agb1-2* and *agg1-1c agg2-1* that exhibit impaired plant immunity in response to P. syringae pathogens. The expression of the SA signaling-related genes *PR1* (*Pathogenesis-related protein 1*), *PR4* (*Pathogenesis-related protein 4*), PAD4 (Phytoalexin Deficient 4) and EDS1 (Enhanced Disease Susceptibility 1) was dramatically increased in wild-type and all G-protein mutants after inoculation with the host pathogen (P. syringae pv. *maculicola*) (Figure 5B; Table S1). Similar results were obtained with plants inoculated with the nonhost pathogen P. syringae pv. *phaseolicola*, although in this case, the transcript levels in *gpa1* and *agg1-1c agg2-1* were slightly less than that in wild-type. In general, these results indicate that the SA defense pathway was not impaired in G-protein mutants upon host and nonhost pathogen infections. 

The expression patterns of JA-signaling pathway-related genes such as *LOX1* (*Lipoxygenase 1*)*, LOX2* (*Lipoxygenase 2*)*, CORI3* (*Coronatine Induced 3*) and AOS (Allene Oxide Synthase) were altered in the G-protein mutants in comparison with wild-type after inoculation with host (P. syringae pv. *maculicola*) and nonhost pathogens (P. syringae pv. *phaseolicola*) (Figure 5B; Table S1). In general, the expression of JA-signaling pathway-related genes was markedly induced at 12 hpi and gradually decreased at 24 hpi in wild-type and G-protein mutants after host pathogen inoculation. The induction was more pronounced in *gpa1-4*, *agb1-2* and *gpa1-4 agb1-2* mutants, suggesting Gα- and Gβ-mediated signaling negatively regulate the JA-related genes. This agrees with the previous report for the role of Gα proteins in regulation of JA signaling pathways [11,45]. In *agg1-1c agg2-1* double mutant, except for *LOX1*, the levels of expression of JA-related genes were similar to that of wild-type, Col-0 (Figure 5B). 

In general, the G-protein mutants showed a substantial reduction in all examined JA-related gene (*LOX1, LOX2, CORI3* and *AOS*) expressions at both 12 and 24 hpi in response to nonhost pathogen infections, except *LOX1* that was markedly induced at 12 hpi in *agg1-1c agg2-1* mutant. *LOX2* was repressed at 12 hpi in all the mutants tested, except *agb1-2* compared to wild-type (Figure 5B). *CORI3* was down-regulated in all mutants tested compared to wild-type. *AOS* expression also decreased in all the mutants tested, compared to wild-type, except for *gpa1-4* and *agg1-1c agg2-1* mutants at 12 hpi. Together our findings suggest that heterotrimeric G-protein-mediated defense pathway against host pathogens may be positively influenced by JA defense signaling in contrast to defense against nonhost pathogens.

Since heterotrimeric G-proteins were induced by both host and nonhost pathogen infections and involved in stomatal defense against bacterial pathogens, we hypothesized that heterotrimeric G-proteins may be involved in *FLS2*-mediated immune response [46]. We monitored the expression of genes known to be associated with *FLS2*-mediated defense such as *RGS1* (regulator of G-protein signaling protein) [47], and *BAK1* (*bri1*-associated receptor kinase) [48,49]. Additionally, because BAK1 appears to have essential role in both brassinosteroid (BR) and flagellin signaling pathways, we also determined the expression changes of BRI1 (Brassinosteroid insensitive 1) after bacterial inoculations. Expression analysis of the *FLS2*-mediated signaling pathway clearly revealed that the level of gene expression was altered to various degrees in response to host and nonhost pathogen in *gpa1-4, agb1-2, gpa1-4 agb1-2* and *agg1-1c agg2-1* mutants (Figure 5B; Table S1). After inoculation with the host pathogen, in general, *BRI1* and *FLS2* were down-regulated in the mutants, while both genes were greatly up-regulated in wild-type at 12 hpi. At 24 hpi, *BRI1* was repressed in wild-type and *gpa1-4*, whereas it was induced in *agb1-*2*, gpa1-4 agb1-2* and *agg1-1c agg2-1* mutants. Interestingly, *RGS1* was significantly down-regulated in *agb1-2* and *gpa1-4 agb1-2* but induced in *agg1-1c agg2-1* compared to wild-type. In response to a nonhost pathogen, the expression levels of the flagellin signaling pathway-related genes examined were similar in all the mutants when compared to wild-type except for *agg1-1c agg2-1* (Figure 5B). These results suggest that the mutation of both Gγ1 and Gγ2 subunits may greatly alter the *FLS2*-mediated defense signaling for bacterial resistance, and support our findings that *agg1-1c agg2-1* double mutant showed the most enhanced disease susceptibility in response to *P. syringe* pathogens. 

*RbohF* (*Respiratory burst oxidase homolog protein F*) has been known for generating reactive oxygen species (ROS) during incompatible interactions with pathogens and is involved in the regulation of stomatal closure and HR related cell death [34,50]. We found that the expression level of *RbohF* was markedly suppressed in all mutants tested at 12 hpi upon nonhost pathogen infection (Figure 5B; Table S1). This result agrees with the recent reports that the H_2_O_2_ level was significantly reduced in *agb1-2* and *agg1-1c agg2-1* mutants [12,34]. OST1 (Opening Stomata 1) is another important gene involved in ABA-induced stomatal closure and guard cell signaling [51]. After host pathogen inoculation, the level of *OST1* expression was significantly elevated at 12 hpi in *gpa1-4, agb1-2, gpa1-4 agb1-2* and *agg1-1c agg2-1* compared to its expression in wild-type. On the other hand, *OST1* gene was down-regulated in *agb1-2, gpa1-4 agb1-2* and *agg1-1c agg2-1* at 24 hpi in response to a nonhost pathogen, suggesting the interaction between *OST1*-mediated guard cell signaling and G-protein signaling.

### Overexpression of genes encoding Gα and Gβ heterotrimeric G-protein subunits partially inhibits growth of host and nonhost pathogens

**Figure 6:**
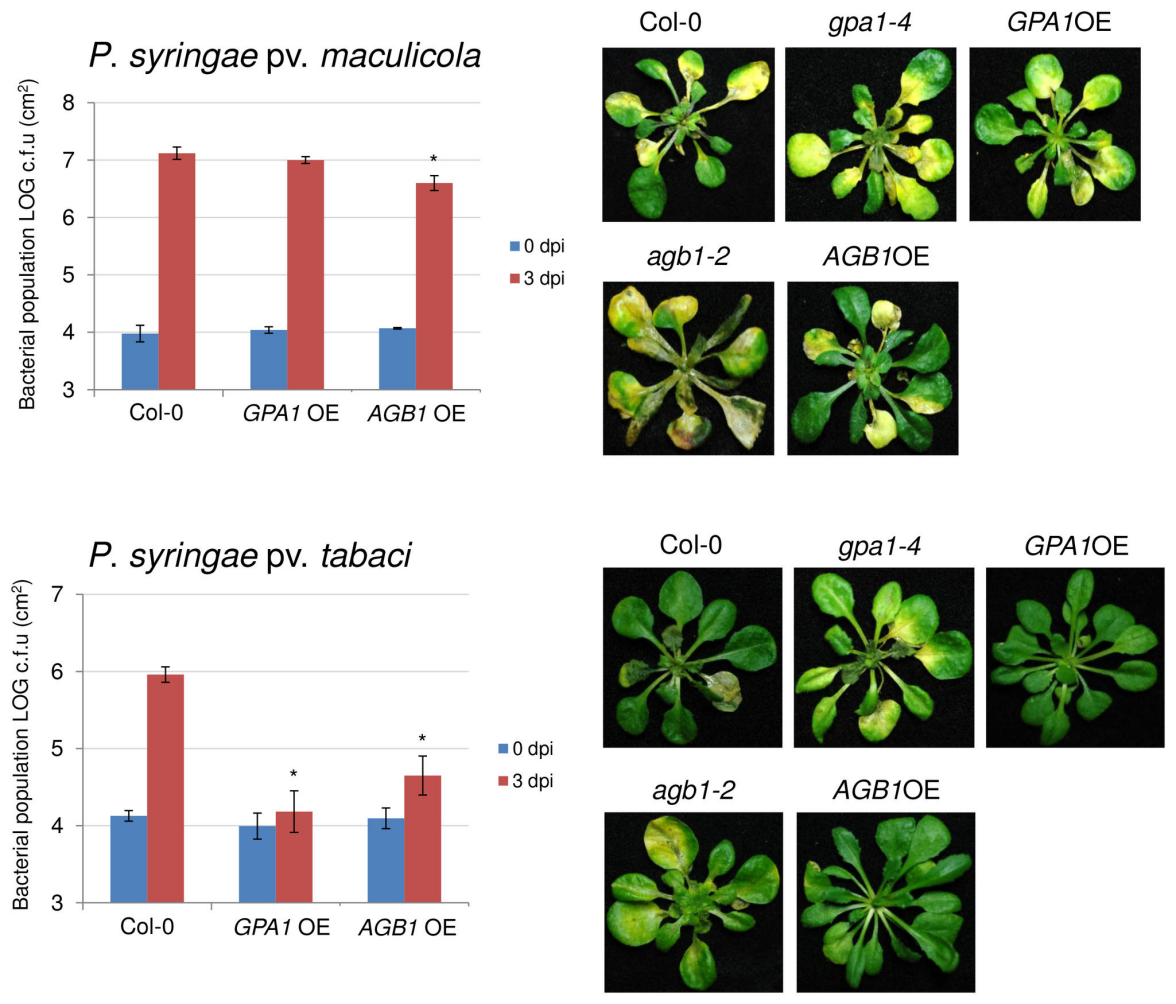
Bacterial growth of P. syringae pv. *maculicola* and P. syringae pv. *tabaci* in wild-type and overexpression lines of GPA1 (GPA1OE) and AGB1 (AGB1OE). Four-week-old plants were flood-inoculated with both pathogens (5×10^7^ CFU/ml). Bacterial titers at 0 and 3 dpi were measured by taking leaf disks from four inoculated plants with three biological replications. All experiments were independently repeated two times. Images for disease phenotypes were taken at 4 dpi. Asterisks above bars represent statistically significant differences in comparison with wild-type plants using Student’s *t*-test (P<0.05).

To determine whether the overexpression of two major G-protein subunits, Gα and Gβ, have any role in bacterial resistance, we tested the transgenic lines overexpressing *GPA1* and *AGB1* (approximately 10 fold changes in transcription level than wild type Col-0) [52] for disease resistance. Overexpression of *GPA1* had no effect on disease resistance to host pathogen, P. syringae pv. *maculicola*; however, *AGB1* overexpressing plants showed slightly reduced bacterial growth three days after P. syringae pv. *maculicola* compared to wild-type plants (Figure 6A). Interestingly, after nonhost pathogen (P. syringae pv. *tabaci*) inoculation, the number of bacterial cells in the leaf apoplast was significantly lower in both *GPA1* and *AGB1* overexpressing plants compared to wild-type (Figure 6B). These results provide further evidence for the role of heterotrimeric G-proteins in plant innate immunity.

## Discussion

Heterotrimeric G-proteins are widely conserved in animals and plants [53,54]. In plants, G-proteins have been shown to regulate important growth and development pathways and ABA signaling [55,56]. There is some evidence for the role of G-proteins in regulation of defense responses in plants; however, a systematic study to characterize the role of G-proteins in plant innate immunity is largely unexplored, especially for bacterial disease resistance. Our findings in the current study clearly demonstrate that the Gα subunit (*GPA1*), Gβ subunit (*AGB1*) and Gγ subunits (*AGG1* and *AGG2*) play a major role in plant innate immunity against P. syringae pathogens. Abolishing expression of these genes disrupts basal, gene-for-gene and nonhost disease resistance against bacterial pathogens in Arabidopsis, showcasing the necessity of signal transduction mediated by *GPA1*-*AGB1*-*AGG1*/*AGG2* (Gα and Gβγ dimer) in defense responses against bacterial pathogens [12,34,35]. 

Heterotrimeric G-proteins are found in all eukaryotic organisms and their role in mediating disease resistance has been established in other organisms as well. In humans, for example, the defects of G-protein signaling can cause an impressive variety of diseases such as mental retardation, cancer, diabetes and congenital bleeding [57–60]. Cholera toxin (CTX) secreted from the bacterial pathogen *Vibrio cholera* targets the Gα subunit and results in malfunctioning of G-protein-mediated pathway [61]. In plants, the involvement of G-proteins in defense was speculated a couple of decades ago [6,62]. Recently, it has been shown that Gα and Gβ subunits of heterotrimeric G-proteins are involved in disease resistance to various fungal and bacterial pathogens in rice and Arabidopsis [10–12,30,34]. Signal transduction mediated by Gα protein has been elucidated in rice [30,63] where it targets established signaling components of disease resistance such as *OsRac1*, important for the production of reactive oxygen species, mitogen-activated protein kinase, *OsMAPK6* [63], the lignin biosynthetic enzyme cinnamoyl-CoA reductase I (*OsCCR1*) which presumably strengthens cell walls [64], and the ROS scavenger metallotionein (*OsMT2b*) that maintains the ROS signaling [65]. In Arabidopsis, the *agb1* mutant which showed enhanced susceptibility to *P*. *cucumerina* was not affected in the production of H_2_O_2_ in response to *P*. *cucumerina* [10]. However, two recent studies demonstrated that the production of H_2_O_2_ was remarkably reduced in the mutants of Gβ and Gγ1Gγ2, suggesting that these G-protein subunits are required for a full oxidative burst in response to *P. syringae* pathogens [12,34]. Torres et al. (2013) demonstrated that *AGB1* is required for resistance in response to *P. syringae* strains, but Gα subunit (*GPA1*) was not found to be involved in bacterial disease resistance in Arabidopsis. However, in this study, we found enhanced disease susceptibility in the *gpa1* mutant against both host and nonhost pathogens. This may be due to the different inoculation methods used in different labs. We used seedling flood-inoculation method that is very sensitive for bacterial disease assay [66] when compared to Torres et al. (2013) that used spray inoculation in adult plants. It has been well characterized that *GPA1* is functionally important for stomatal closure in response to abiotic and biotic stresses [6,14,67]. Due to the loss of stomatal defense in *gpa1* mutant, a large number of bacterial cells can enter through stomata (Figure 4) and may enhance disease symptom development in Arabidopsis seedlings. Moreover, it has been demonstrated that the mutation of Gα subunit reduced H_2_O_2_ production and *PR* gene expression upon blast pathogen infection in rice, indicating the important role of Gα in plant defense signaling [30]. 

 The Gγ subunit is an important part for the heterotrimer that binds to Gβ and anchors the Gβγ dimer to the plasma membrane [31,68]. Gβγ dimer is the active signaling entity in many physiological processes similar to the Gα subunit [33,52,69]. The resistance to fungal pathogens such as *F. oxysporum*, *A. brassicicola*, *B. cinerea* and *P. cucumerina* was impaired in *agb1* and *agg1* mutant plants [8,10,11,31]. As we have shown in this study, *AGG1* and *AGG2* play redundant functions in the regulation of Arabidopsis defense response to *P. syringae* pathogens; whereas no obvious roles were observed for the *AGG3*. Liu et al. (2013) also recently demonstrated that *AGG1* and *AGG2* play an important role for cell death and PAMP triggered immunity in Arabidopsis. In Arabidopsis, Gγ subunits are solely responsible for any functional specificity of G-protein heterotrimer, and it is likely that the AGG1 and AGG2 proteins are, in general, involved in regulating biotic stress-related signaling pathways, whereas AGG3 is mostly involved in regulation of abiotic stresses [70,71]. Our gene expression result also supports that *AGG3* was not induced upon host and nonhost pathogen infections (Figure 5A). Analysis of additional higher order mutants, such as, GαGβGγ_1_, GαGβGγ_2_, GαGβGγ_3_, Gγ_1_Gγ_2_Gγ_3_, in the future would provide further insight to the role of specific G-protein subunit combinations in controlling signal-response coupling. 

Recent studies provide evidence that plant stomata can play an active role in restricting bacterial invasion as part of the plant innate immune system [41]. Perception of multiple bacterial PAMPs, including flagellin, lipopolysaccharide (LPS) and nonhost bacterial pathogens, induce closure of stomata in epidermal peels of *Arabidopsis* leaves [37,41,72–75]. Additionally, we previously showed that nonhost bacteria also induce closure of stomata [74]. In this study, a significant inhibition of stomatal closure was observed after two hrs of contact with both host and nonhost bacterial pathogens in Gα- and Gβ-deficient mutants but not in Gγ-deficient mutant (Figure 4). This result agrees with the previous findings that *gpa1* and *agb1* mutants are hyposensitive to ABA-mediated inhibition of stomatal opening [8,39,76] and suggest that guard cells have developed G-protein-mediated defense mechanisms to control stomatal aperture in response to bacterial pathogens. The lack of this phenotype in single or double *agg1-1c agg2-1* mutants is surprising, and suggests that for this specific response, all three Gγ proteins might be required. Analysis of *agg1agg2agg3* triple mutants, when they become available, will be able to solve this enigma. 

It has been well known that GPA1 is involved in many physiological responses and plant hormonal signaling, including abscisic acid (ABA), gibberellic acid (GA) and brassinosteroid (BR). GPA1 affects ABA signaling and impairs closure of stomata in response to ABA [51,67]. Recently, several studies have also shown that Gα participates in brassinosteroid (BR) responses in *Arabidopsis* and rice plants [77,78]. Rice Gα affects the BR signaling cascade, but the Gα subunit is not a signaling molecule in the BRI1-mediated signaling pathway [78]. In Arabidopsis, BR regulates plant immunity at multiple levels. BR induces BRI1 binding to BAK1 and can suppress MAMP-triggered immunity (MTI) through an unknown mechanism downstream of BIK1 [79,80]. It has also been known that the FLS2 and the heterotrimeric G-protein GPA1 recognize bacterial flagellin to reduce bacterial invasion through stomata in the epidermis and bacterial multiplication in the apoplast [14,33,81]. *fls2* and *gpa1* mutant plants are more susceptible to *Arabidopsis* host pathogen *Pst DC3000* [14]. Our result also shows that *BAK1* is greatly down-regulated in *gpa1-4* and *agg1-1c agg2-1* at 24 hrs after host and nonhost pathogen infections (Figure 5B). In addition, Liu et al. (2013) also described that heterotrimeric G-proteins are involved in the defense signal pathway mediated by the receptor-like kinase (RLK) *SOBIR1* (*suppressor of bir1-1*). 

Another ABA signaling component, OST1, is also required for bacteria- and PAMP-induced stomatal closure [41,76]. In general, it was proposed that GPA1 and OST1 function in the guard cell ABA signaling pathways downstream of PAMP perception. However, the signaling for FLS2-mediated stomatal closure induced by bacteria and PAMPs remains unclear. As shown in Figure 5, the gene expression profiling data suggest that heterotrimeric G-proteins-mediated defense signaling is closely connected to FLS2-mediated immunity through differential expression of several key genes for the pathway such as *RGS1*, *BAK1*, and *BRI1*. Moreover, our finding suggests that GPA1, OST1 and FLS2 may be functionally connected together for guard cell signaling and bacterial disease resistance. 

We observed significant changes in the expression of JA-related genes in response to host and nonhost bacterial pathogen infections (Figure 5b). It is well known that JA signaling antagonizes SA-dependent defense pathway that confers resistance to *Pseudomonas syringae* pathogens. For example, the loss of JA signaling in the coronatine-insensitive 1 (*coi1*) mutant sensitized the SA defense pathway and thus conferred enhanced resistance to bacterial pathogens [82]. As expected, the expressions of *LOX1*, *LOX2*, *CORI3*, and *AOS* were rapidly increased in response to host pathogen and were much higher in all G-protein mutants tested when compared to the wild-type plants (Figure 5). Interestingly, however, the expression of JA-related genes was generally down-regulated in response to nonhost pathogen, suggesting an independent negative role of JA signaling for heterotrimeric G-protein-mediated nonhost resistance. The expression patterns between *LOX1* and *LOX2* were different, which is well supported by previous studies involving JA signaling during leaf senescence [83]. *LOX2* plays a role in wounding- and defense-related response whereas *LOX1* is strongly up-regulated during leaf senescence in Arabidopsis [84]. The level of *LOX2* expression was greatly down-regulated at 24 hpi in all mutants tested except *agb1-2*, indicating that AGB1-mediated defense pathway may be not be same as GPA1- and AGG1 AGG2-mediated bacterial defense signaling. 

**Figure 7:**
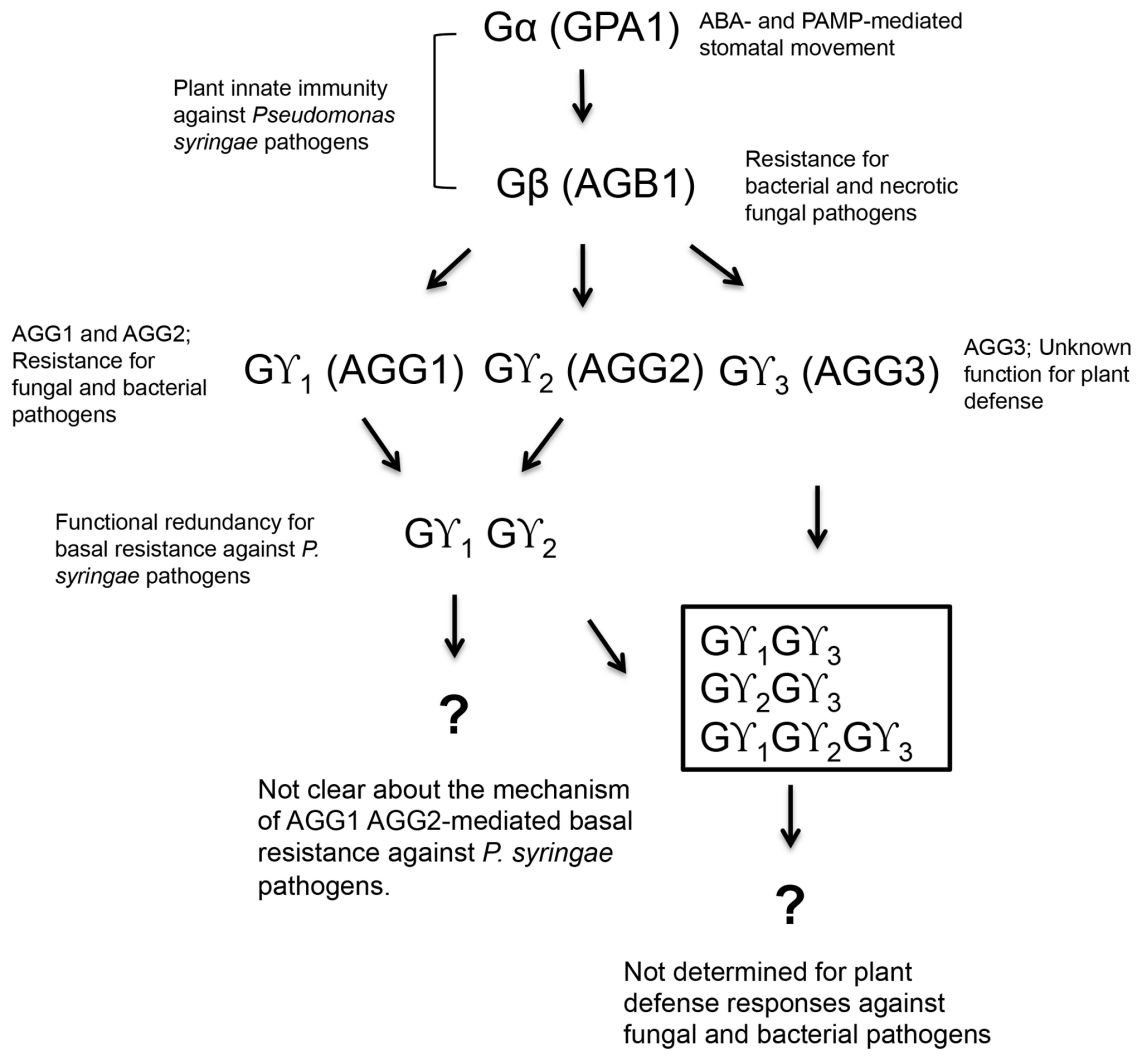
Proposed model for the plant innate immunity signaling network regulated by heterotrimeric G-proteins in Arabidopsis.

 Based on our results and previous reports, we propose a working model for the mechanism of G-protein-mediated plant immunity in response to *P. syringae* pathogens (Figure 7). The Arabidopsis Gα regulates early defense responses, including stomatal closure, ROS production and cell death progression in response to ABA, ozone, bacteria and PAMPs [67,85,86]. We also found the expression pattern of *RbohF* that is involved in ROS production was altered in *agb1* and *agg1-1c agg2-1* mutants (Figure 5B). It has been shown that the Gα-deficient mutant is slightly more resistant to necrotrophic fungal pathogens than wild-type plants [10,11]. In contrast, the *agb1* mutant is more susceptible than wild-type plants to necrotrophic and vascular fungal pathogens [10,11]. The double mutant of *gpa1-4 agb1-2* was as susceptible as *gpa1* and *agb1* single mutants, and did not indicate any additive effect. Contrarily, the *agg1-1c agg2-1* double mutant displayed enhanced disease susceptibility to host and nonhost pathogens, suggesting that either Gβγ1 or Gβγ2 is necessary for the specific dimer involved in the regulation of plant immunity against *P. syringae* pathogens. Thus, *AGG1* and *AGG2* have redundant functions for bacterial defense responses, while the Gβγ1 dimer (*AGB1* and *AGG1*) is only required for the immune response against necrotrophic fungi in Arabidopsis [8,11]. This suggests that the involvement of heterotrimeric G-proteins in plant immunity can vary depending on the plant species and the pathogens studied. Moreover, it would be interesting to examine whether *AGG3* (or Gβγ3 dimer) is involved in bacterial resistance and if there are any phenotypic changes in Gγ1Gγ3-, Gγ2Gγ3- or Gγ1Gγ2Gγ3-deficient mutants. More importantly, future studies for identifying Gγ1Gγ2 (or Gβγ dimer) targets and characterizing their downstream signaling will be needed to understand the entire pathway of G-protein-mediated plant innate immunity against bacterial pathogens.

## Materials and Methods

### Plant materials and bacterial pathogens

Wild-type Arabidopsis Columbia (Col-0) and T-DNA knockout mutants for heterotrimeric G-protein subunits, *gpa1-4* (SALK_001846), *agb1-2* (CS6536), *gpa1-4 agb1-2* (CS6535), *agg1-2* (GABI: accession no. 736A08), *agg2-2* (Nottingham Arabidopsis Science Centre: accession no. N375172), *agg1-1c agg2-1* (CS16551), and *agg3-2* (CS807967) were used in this study. All the G-protein mutants tested were in Col-0 background and expressed the *RPM1* gene. *agg1-1c* is originally from WS-0 containing the natural mutation (premature stop) at the kinase domain of *FLS2*, and the original *agg1-1c* in WS-0 was backcrossed nine times to Col-0 [31,87]. We sequenced RT-PCR product of *FLS2* from *agg1-1c agg2-1*mutant. The *FLS2* sequence of *agg1-1c agg2-1* was same as the full length *FLS2* sequence from Col-0, indicating *FLS2* is fully functional in *agg1-1c agg2-1*. The overexpression lines for *GPA1* and *AGB1* were obtained from Dr. Alan Jones (University of North Carolina, Chapel Hill, NC) [52]. Phenotypes of each of the mutants are shown in Figure S1. Confirmation of gene knock-out was done by semi-quantitative RT-PCR (Figure S1). Wild-type and G-protein mutant seedlings were germinated on 1/2 strength Murashige and Skoog (MS) agar medium and transferred to 1/2 strength MS plates or soil for further experiments. For seedling flood-inoculation [88], four Arabidopsis plants were grown in individual 1/2 strength MS plate under short-day conditions (10 hrs light/14 hrs dark) in a controlled environment chamber at 25° C for three weeks and three plates were used for the inoculation of each pathogen. For syringe-inoculation and other experiments, the plants were transferred to soil and grown for four weeks in a growth chamber at 20° C to 22° C under 8 hrs light/16 hrs dark regime. 

Bacterial pathogens P. syringae pv. *maculicola*, P. syringae pv. *tabaci*, P. syringae pv. *phaseolicola*, P. syringae pv. *maculicola* (*AvrRpm1*) were grown overnight at 28° C in King’s B (KB) medium containing appropriate antibiotics at the following concentrations (μg ml^−1^): rifampicin, 50 and kanamycin, 25. Bacterial cultures were centrifuged at 3,000 rpm for 10 min, and the cell pellet was suspended in sterile distilled water; OD_600_ was measured and bacterial titer was adjusted depending on the assay. 

### Bacterial pathogen inoculations

For flood-inoculation, 4-week-old plants grown in 1/2 strength MS plates were incubated for 5 minutes with 40 ml of bacterial suspension at a final concentration of 3×10^6^ CFU/ml. At defined time points, inoculated leaves were harvested, ground and serially diluted as described [88]. For syringe inoculation, 6-week-old plants were infiltrated with a needleless syringe on the abaxial side of the leaves with bacterial pathogens at a concentration of 1×10^4^ CFU/ml. Inoculated leaves were collected at different time points and used to examine bacterial growth. 

### Stomata assay

Stomatal closure assay was performed according to published protocols [41,89]. To assure that most stomata are open before beginning of the experiments, plants were conditioned under light for at least 3 hrs, and detached epidermal peels were immediately floated on stomatal opening buffer (10 mM MES-Tris; 10 mM KCl, pH 6.3) for 2 hrs. The epidermal peels were further incubated for 2 hrs in the presence of nonhost bacterial pathogen *P. syringae* pv. *tabaci* (6×10^8^ CFU/ml). The width and length of stomatal pores were measured by observing approximately 30 stomata in each epidermal peel. A total of six epidermal peels per genotype were examined for each treatment.

The numbers of bacterial cells that entered through stomata were measured using 2-week-old seedlings grown in 1/2 strength MS medium. P. syringae pv. *tabaci* was grown in KB medium overnight at 28° C, centrifuged at 3,500 rpm for 10 min and resuspended in sterile distilled water at a concentration of 1×10^7^ CFU/ml. Detached Arabidopsis leaves were floated on bacterial suspension (cuticle leaf surface was in contact with bacterial suspension). After 2- or 4-hr incubation, the leaf surface was sterilized using 10% bleach (Clorox) and plated on KB medium to measure the number of bacterial cells in apoplast. This experiment was repeated three times under the same conditions. 

### Assay of real-time quantitative reverse transcriptase PCR (qRT-PCR)

Total RNA was extracted from Arabidopsis leaves infiltrated with water (mock control), host pathogen (*P. syringae* pv. *maculicola*) or nonhost pathogen (*P. syringae* pv. *phaseolicola*), sampled at 0, 12 and 24 hrs post-inoculation (hpi). RNA samples were treated with DNAseI (Ambion, Austin, TX) and used for cDNA synthesis using SuperScript III reverse transcriptase (Invitrogen, Grand Island, NY, USA). The cDNA was diluted to 1:20 and used for qRT-PCR using Power SYBR Green PCR master mix (Applied Biosystems, Foster City, CA, USA) with an ABI Prism 7900 HT sequence detection system (Applied Biosystems, Foster City, CA, USA). Amplification of Arabidopsis *Ubiquitin 5* (*UBQ5*) and *Elongation factor 1α* (*EF1α*) was used as internal control to ensure an equal amount of cDNA in individual reactions. To determine the role of the SA-mediated and JA-mediated hormonal pathways and *FLS2*-mediated defense in G-protein-mediated signaling, qRT-PCR was performed with primers designed for amplifying *PR1, PR5, PAD4, EDS1, CORI3, AOS, LOX1, LOX2, OST1, RbohF, RGS1, FLS2, BAK1* and *BRI1* genes (Table S2). Two biological replicates of each sample and three technical replicates of each biological replicate were used for qRT-PCR analysis. Average Cycle Threshold (Ct) values, calculated using Sequence Detection Systems (version 2.2.2; Applied Biosystems) from all the replicates per sample were used to determine the fold expression relative to controls.

## Supporting Information

Table S1 **Heat map of transcript accumulation of heterotrimeric G-proteins and defense related genes (SA-, JA- and *FLS2*-mediated defense signaling) in response to P. syringae pv. *maculicola* and P. syringae pv. phaseolicola infections.** This is the same heat map data as shown in Figure 5, the actual numbers of fold changes are included in each column.
(XLSX)

Table S2 **List of primers used for semi-quantitative PCR and real-time PCR.** Primers for *GPA1*, *AGB1*, *AGG1*, *AGG2*, and *AGG3* were used for semi-quantitative PCR (named as GPA1-semiRT, AGB1-semiRT, AGG1-semiRT, AGG2-semiRT, and AGG3-semiRT).
(XLSX)

Figure S1 **Plant growth patterns of heterotrimeric G-protein mutants and determination of null mutation by RT-PCR.** Arabidopsis seedlings were grown in 1/2 strength MS for four weeks at 20 to 23° C (10 hrs daylight). Total RNA was isolated from wild-type Col-0 and heterotrimeric G-protein mutants and analyzed by RT-PCR using gene specific primers for *GPA1*, *AGB1*, *AGG1*, *AGG2* and *AGG3*.
(TIF)
