# Supplementary figures and images for: Arabidopsis Heterotrimeric G-Proteins Play a Critical Role in Host and Nonhost Resistance against Pseudomonas syringae Pathogens
Source: PLoS One. 2013 Dec 5;8(12):e82445. doi: 10.1371/journal.pone.0082445 (PMC3857812; doi:10.1371/journal.pone.0082445)

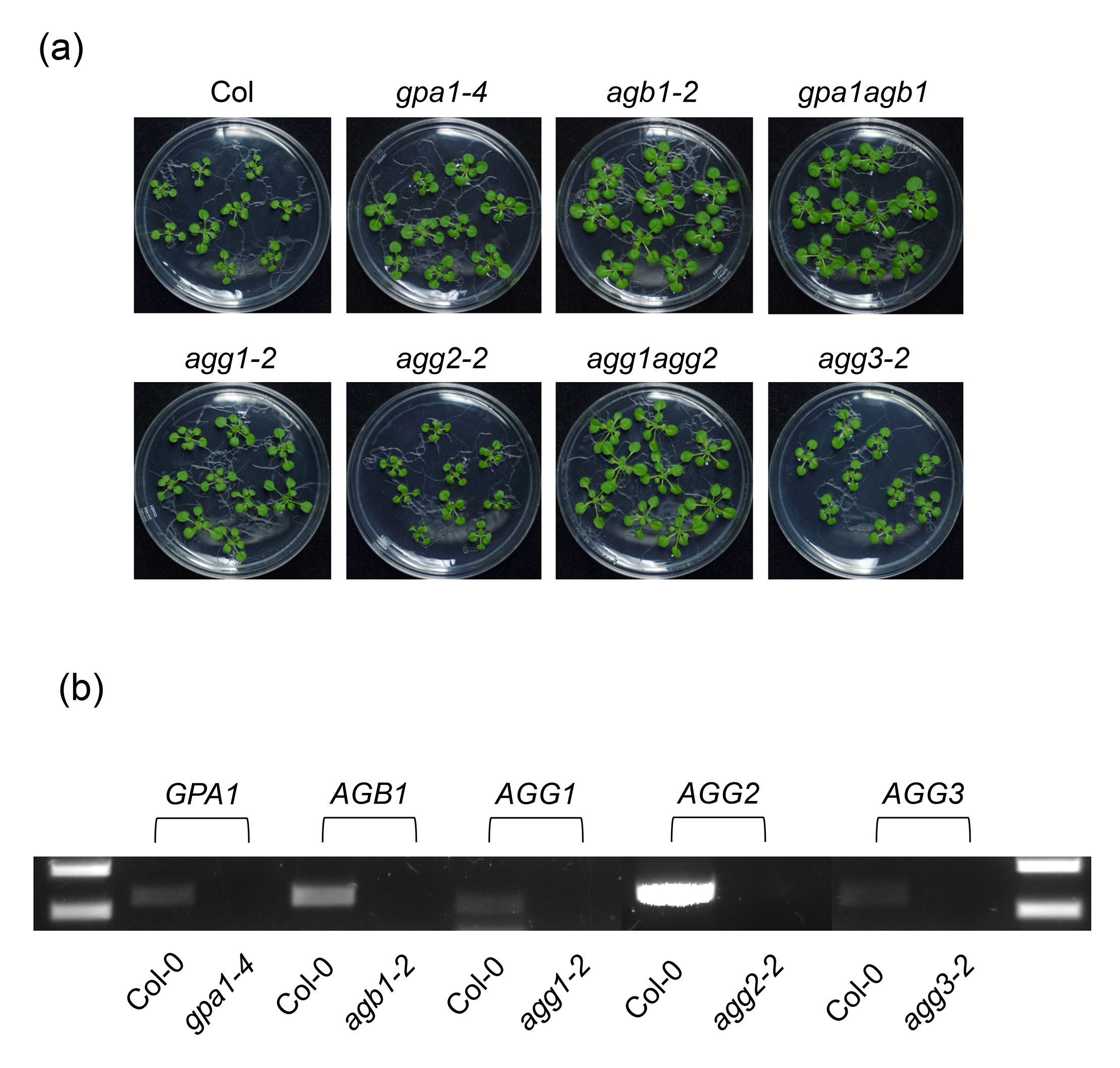

Supplement: Figure S1 — Plant growth patterns of heterotrimeric G-protein mutants and determination of null mutation by RT-PCR. Arabidopsis seedlings were grown in 1/2 strength MS for four weeks at 20 to 23° C (10 hrs daylight). Total RNA was isolated from wild-type Col-0 and heterotrimeric G-protein mutants and analyzed by RT-PCR using gene specific primers for GPA1, AGB1, AGG1, AGG2 and AGG3. (TIF) [file pone.0082445.s003.tif]
